# THE RELATIONSHIP BETWEEN DAY SERVICE UTILIZATION AND THE CARE LEVEL DETERIORATION FOR PEOPLE WITH DEMENTIA

**DOI:** 10.1093/geroni/igae098.2851

**Published:** 2024-12-31

**Authors:** Liu Yang, Yamakawa Miyae, Sugiura Aki, Kanaya Reiko, Wu Yunfan, Yasushi Takeya

**Affiliations:** Osaka University, Suita, Osaka, Japan; Osaka University, Suita, Osaka, Japan; Osaka University, Suita, Osaka, Japan; Osaka University, Suita, Osaka, Japan; Osaka University, Suita, Osaka, Japan; Gerontological and Geriatric Nursing Research Unit, Osaka, Osaka, Japan

## Abstract

In Japan, people diagnosed with dementia are often recommended to use day services (DS). This study aims to evaluate whether DS has a positive impact on preserving the independence of people with dementia in their daily lives.We analyzed 774 individuals aged 65-74, initially certified as care level 2 or below, between 2012 and 2019 from Osaka Prefecture National Health Insurance Database. The baseline was set three years after their initial certification, and the outcome measure was reaching level 3 or higher care levels. This retrospective cohort study categorized subjects into three groups: Group 1 (non-DS users during the exposure period), Group 2 (DS users for over one but less than two years continuously), and Group 3 (DS users for two years or more). Median survival time was not observed for Groups 1 and 2, while Group 3 had a median survival time of 921 days (95% CI 668-1174 days), showing a statistically significant difference among the three groups (Log Rank P< 0.000). In Cox analysis, the presence of fractures (HR = 2.33; 95% CI = 1.07–5.05, P = 0.032) and baseline care level (HR = 2.54; 95% CI = 1.98–3.28, P < 0.000) demonstrated positive correlations with the outcome measure. The results indicate that the use of DS does not effectively slow down the deterioration of care levels in people with dementia. The independence in daily life for people with dementia does not show a positive impact after using DS.

